# Long-term motor stability but increasing non-motor burden in multifocal motor neuropathy: a 6-year real-world follow-up study during the COVID-19 era

**DOI:** 10.3389/fnhum.2026.1824742

**Published:** 2026-05-08

**Authors:** Ivana Basta, Marta Jeremic, Aleksa Palibrk, Nikola Momcilovic, Vukan Ivanovic, Mladen Jankovic, Nenad Jovanovic, Ivana Bulatovic, Stojan Peric, Ivo Bozovic

**Affiliations:** 1 University Clinical Center of Serbia-Neurology Clinic, Belgrade, Serbia; 2 University of Belgrade-Faculty of Medicine, Belgrade, Serbia; 3 Neurology Clinic, University Clinical Center of Montenegro, Podgorica, Montenegro

**Keywords:** multifocal motor neuropathy (MMN), longitudinal study, quality of life, COVID-19, depression, fatigue

## Abstract

**Introduction:**

Multifocal motor neuropathy (MMN) is a chronic immune-mediated disorder associated with long-term disability and reduced quality of life (QoL). Longitudinal real-world data on QoL in MMN are scarce, particularly during the COVID-19 era. This study aimed to evaluate long-term changes in QoL and disease outcomes in MMN patients over a 6-year follow-up and to assess the multidimensional impact of the COVID-19 pandemic.

**Methods:**

Twelve MMN patients were re-evaluated 6 years after baseline assessment. Functional disability was assessed using the INCAT disability score and I-RODS scale. QoL was measured using the SF-36 questionnaire, while depressive symptoms and fatigue were evaluated using the Beck Depression Inventory (BDI) and Fatigue Severity Scale (FSS). Pandemic-related impact was assessed using a specifically designed questionnaire.

**Results:**

Functional disability remained overall stable during follow-up (INCAT total score 3.4 ± 2.2 vs. 3.3 ± 1.9). However, fatigue and depressive symptoms increased significantly, with the prevalence of fatigue rising from 17 to 58% and depression from 8 to 25% (*p* < 0.05). Mean FSS scores increased from 20.1 ± 13.3 to 35.3 ± 15.0, and BDI scores from 3.3 ± 7.8 to 9.2 ± 15.4. Physical QoL domains declined over time, particularly Physical Functioning (76.7 ± 29.9 to 57.5 ± 31.6) and Bodily Pain (82.8 ± 28.9 to 71.7 ± 28.5), while mental domains remained relatively preserved (MCS 69.1 ± 19.5 vs. 69.4 ± 21.3). During the COVID-19 pandemic, 33% of patients developed SARS-CoV-2 infection, 17% experienced treatment interruption, and one patient died from severe COVID-19.

**Conclusion:**

Long-term immunotherapy in MMN appears to stabilize motor disability but does not prevent the progressive increase of fatigue and depressive symptoms, which emerge as major contributors to long-term disease burden. The COVID-19 pandemic further affected healthcare access and psychosocial wellbeing. These findings support routine screening for fatigue and mental health symptoms and highlight the need for a multidisciplinary approach to MMN management.

## Introduction

Multifocal motor neuropathy (MMN) is an immune-mediated disorder characterized with asymmetric and progressive distal muscle weakness, combined with the absence of sensory symptoms (Lawson and Arnold, 2014). MMN as a chronic disease with progressive course can significantly impact patients’ quality of life (QoL). Previous studies have indeed shown that patients with MMN experience a significant reduction in quality of life, particularly but not exclusively regarding the physical domains (Bozovic et al., 2019; Stangel et al., 2016; Misbah et al., 2011; Harbo et al., 2009; Eftimov et al., 2009). Current clinical practice favors intravenous immunoglobulins as the treatment of choice for MMN, with randomized clinical trials indicating improvement of muscle strength and functional disability in these patients (Bozovic et al., 2019; Stangel et al., 2016; Misbah et al., 2011; Harbo et al., 2009; Eftimov et al., 2009; Katzberg et al., 2016). However, it seems that even treated MMN can chronically affect QoL of these patients and may lead to an unfavorable outcome. Nevertheless, most of the available literature has assessed quality of life in MMN mainly in cross-sectional settings or in the context of treatment response in clinical trials (Bozovic et al., 2019; Stangel et al., 2016; Misbah et al., 2011; Harbo et al., 2009; Eftimov et al., 2009). Although reduced quality of life, particularly in physical domains, has been reported in MMN, longitudinal real-world data on the evolution of disability, fatigue, depressive symptoms, and quality of life remain scarce. In particular, the long-term burden of MMN in the context of the COVID-19 era has not been sufficiently characterized.

The recently emerged COVID-19 disease is a rapidly transmittable viral infection caused by a novel coronavirus, known as severe acute respiratory syndrome coronavirus 2 (SARS-CoV-2) (Seyed Hosseini et al., 2020). It might be supposed that both treated and untreated patients with an immune-mediated neuropathy such as multifocal motor neuropathy could represent an especially vulnerable population. On the other hand, several studies have already underlined the influence of COVID-19 pandemic on patients’ mental health. Indeed, it seems that patients with different neurological autoimmune disorders have been more frequently, additionally diagnosed with depression, insomnia or anxiety during the COVID-19 pandemic (Stojanov et al., 2021; Peric et al., 2023; Taga and Lauria, 2022; Pugliatti et al., 2022). However, there is still insufficient data about the influence of COVID-19 pandemic on both mental (such as increased rates of anxiety, depression, sleep disturbance) and physical aspects of QoL and disease outcome in MMN patients.

Beyond the acute infection, post-COVID conditions may include persistent fatigue, reduced physical endurance, and cognitive complaints commonly described as “brain fog.” In this context, brain fog refers to difficulties with attention, concentration, memory, and mental clarity that may interfere with daily functioning and quality of life. Fatigue and cognitive impairment are among the most frequently reported long-term manifestations after COVID-19 (Graham et al., 2021; Ceban et al., 2022). Emerging rehabilitation approaches further support the clinical relevance of these symptoms, including a pilot study of a dual-task augmented reality protocol in adults with self-reported long COVID fatigue and cognitive impairment, as well as a randomized trial protocol investigating resistance exercise after COVID-19 infection (Deodato et al., 2024; Morrow et al., 2022). However, the possible contribution of pandemic-related burden and post-COVID symptoms to long-term patient-reported outcomes in MMN has not been sufficiently explored.

The primary aim of this longitudinal study was to evaluate changes in quality of life, disability, fatigue, and depressive symptoms in a cohort of patients with multifocal motor neuropathy over a 6-year follow-up period. The secondary aim was to assess the multidimensional impact of the COVID-19 pandemic on this cohort.

## Materials and methods

This was a single-center longitudinal observational follow-up study of a previously characterized cohort of patients with MMN, initially assessed in 2017 at the Neurology Clinic, University Clinical Center of Serbia (Bozovic et al., 2019). At 6-year follow-up, all available patients from the original cohort were invited for re-evaluation during routine neurological visits. During the 6-year follow-up period, 12 of the 17 patients evaluated at baseline were re-assessed. Two patients died, and three were lost to follow-up or declined participation. Only patients fulfilling the European Federation of Neurological Societies/Peripheral Nerve Society (EFNS/PNS) diagnostic criteria for MMN were included in this study (Van Schaik et al., 2010).

Given the rarity of multifocal motor neuropathy, the study sample was based on all eligible patients from the original cohort who were available for follow-up assessment, and no formal *a priori* sample size calculation was performed. Therefore, the study should be considered exploratory, and the limited sample size should be taken into account when interpreting between-timepoint comparisons and multivariable analyses. The study was approved by the Ethics Committee of the University Clinical Center of Serbia and the Ethics Committee of the University of Belgrade – Faculty of Medicine, and all participants provided written informed consent.

Sociodemographic and clinical data (including gender, age, education, employment status, disease duration and functional disability) were collected at the time of follow-up testing. All therapeutic modalities (including concomitant medication) and treatment responses were recorded, as well as the presence of relevant comorbidities.

Functional disability was assessed using established disability scales for inflammatory neuropathies. The Inflammatory Neuropathy Cause and Treatment (INCAT) disability score and the Inflammatory Rasch-built Overall Disability Scale (I-RODS) were used at both baseline and follow-up (Breiner et al., 2014; Draak et al., 2016). Disease worsening was defined as an increase of at least 1 point on the adjusted INCAT disability scale, whereas disease improvement was defined as a decrease of at least 1 point (Breiner et al., 2014). The I-RODS consists of 24 activities graded from easy to difficult, with each item scored as not possible (0), possible with difficulty (1), or possible without difficulty (2), yielding a total score ranging from 0 to 48, with higher scores indicating better functioning and social participation (Draak et al., 2016). In addition, at follow-up, the Rasch-built Overall Disability Scale for MMN (MMN-RODS) was applied as a disease-specific measure of activity limitation in MMN patients (Vanhoutte et al., 2015).

Because depressive symptoms and fatigue are recognized contributors to long-term disease burden and reduced quality of life in chronic neurological disorders, these domains were additionally assessed using the Beck Depression Inventory (BDI), with a cut-off value of ≥11 indicating depression, and the Fatigue Severity Scale (FSS), with scores ≥36 indicating severe fatigue (Beck et al., 1988; Krupp et al., 1989).

To evaluate health-related QoL, all patients completed the Serbian version of the SF-36 questionnaire (Ware and Sherbourne, 1992), a self-reported instrument comprising eight health domains: physical functioning (PF), role physical (RP), bodily pain (BP), general health (GH), vitality (VT), social functioning (SF), role emotional (RE), and mental health (MH). Together with the total SF-36 score, the Physical Composite Score (PCS) and Mental Composite Score (MCS) summarize these domains. All scores were assessed on a 0–100 scale, with higher values indicating better quality of life.

To assess the multidimensional impact of the COVID-19 pandemic, patients completed a specifically designed 22-item questionnaire covering SARS-CoV-2 infection, access to neurological care, treatment interruption, limitations in daily activities, disease worsening, and psychological concerns related to the pandemic. Because no standardized instrument was available at study initiation to evaluate the pandemic-related burden in MMN, a study-specific questionnaire was used.

### Statistical analysis

Data distribution was assessed using the Kolmogorov–Smirnov test. Continuous variables are presented as mean ± standard deviation or median (range), as appropriate, and categorical variables as counts and percentages. Within-subject comparisons between baseline and 6-year follow-up were performed using the paired Student’s t-test or Wilcoxon signed-rank test, depending on data distribution. Comparisons between independent groups were performed using the Mann–Whitney U test or χ^2^ test, as appropriate. Associations between variables were assessed using Spearman’s rank correlation coefficient. A *p* value <0.05 was considered statistically significant.

## Results

The main sociodemographic and clinical characteristics of the study cohort are presented in Table 1. The final sample comprised 12 patients, of whom 8 (66.7%) were male. At follow-up, mean age was 44.7 ± 11.2 years, and mean disease duration was 11.4 ± 4.9 years.

**Table 1:** Demographic and clinical characteristics of patients with MMN at baseline and 6-year follow-up.

Features	Baseline (*N* = 17)	Follow-up (*N* = 12)
Male gender n(%)	12 (71%)	8 (66.7%)
Age (years, mean ± SD)	37.8 ± 12.8	44.7 ± 11.2
INCAT arms disability score (mean ± SD)	2.7 ± 1.2	2.5 ± 0.7
INCAT legs disability score (mean ± SD)	0.6 ± 1.3	0.83 ± 1.4
INCAT total disability score (mean ± SD)	3.4 ± 2.2	3.33 ± 1.9
I-RODS—centile score (mean ± SD)	59.0 ± 28.5	65.4 ± 23.7
MMN-RODS median (range)	NA	68 (0–94)
FSS (mean ± SD)	20.1 ± 13.3*	35.3 ± 15.0*
Fatigue n(%)	2 (16.7%)	7 (58.3%)
BDI (mean ± SD)	3.3 ± 7.8*	9.2 ± 15.4*
Depression n(%)	1 (8.3%)	3 (25%)

INCAT, Inflammatory Neuropathy Cause and Treatment score; I-RODS, Inflammatory Rasch-built Overall Disability scale; FSS, Fatigue Severity Scale; BDI, Beck’s Depression Inventory; NA, not applicable; *p < 0.05 compared with baseline.

The total Inflammatory Neuropathy Cause and Treatment disability score at follow-up testing (3.3 ± 1.9) remained overall comparable to baseline values (3.4 ± 2.2), suggesting relative stabilization of functional disability during long-term immunotherapy. A non-significant improvement in I-RODS centile score was observed at follow-up testing compared with baseline (65.4 ± 23.7 vs. 59.0 ± 28.5).

Fatigue and depressive symptoms became substantially more prevalent over time (17% vs. 58 and 8% vs. 25%, respectively). Mean FSS score increased significantly (20.1 ± 13.3 to 35.3 ± 15.0, *p* < 0.05), as did BDI score (3.3 ± 7.8 to 9.2 ± 15.4, *p* < 0.05).

Results on the SF-36 scale are shown in Table 2. Physical quality of life domains remained more affected than mental domains (PCS 60.87 ± 24.87 vs. MCS 69.39 ± 21.27). The most impaired domains were Physical Functioning and Role Physical, while Mental Health and Social Functioning remained relatively preserved. Bodily Pain and Physical Functioning showed a statistically significant decrease during follow-up, whereas General Health and Social Functioning improved.

**Table 2:** Quality of life measured by the SF-36 questionnaire at baseline and at 6-year follow-up.

SF-36 scale	Baseline score (*N* = 17)	Follow-up score (*N* = 12)
Physical functioning (PF)	76.7 ± 29.9*	57.5 ± 31.6*
Role physical (RP)	62.5 ± 44.6	45.8 ± 43.7
Bodily pain (BP)	82.8 ± 28.9*	71.7 ± 28.5*
General health (GH)	48.8 ± 24.8	63.8 ± 23.5
Vitality (VT)	65.4 ± 17.9	65.4 ± 21.7
Social functioning (SF)	71.9 ± 36.9	84.4 ± 15.2
Role emotional (RE)	80.5 ± 38.8	58.3 ± 45.2
Mental health (MH)	78.7 ± 17.6	75.0 ± 17.1
Physical composite score (PCS)	67.2 ± 22.1	60.9 ± 24.9
Mental composite score (MCS)	69.1 ± 19.5	69.4 ± 21.3
Total SF-36 score	70.9 ± 21.1	65.2 ± 23.3

* Significant change from baseline (*p* < 0.05). All values are presented as mean ± SD.

Variables associated with quality of life in univariate analyses were entered into a multiple linear regression model. However, no statistically significant independent predictors were identified.

Main results of the specifically designed questionnaire assessing the impact of the COVID-19 pandemic showed that approximately 42% (5/12) of patients reported concerns regarding the potential influence of the pandemic on their disease. One third of patients (4/12) were unable to attend regular neurological check-ups, and about one quarter (3/12) reported limitations in daily activities related to COVID-19. Disease worsening was observed in one patient (1/12; 9%), and MMN treatment was interrupted in two patients (2/12; 17%) due to organizational difficulties during the pandemic.

Four patients (4/12; 33%) developed SARS-CoV-2 infection. Three patients had mild COVID-19 treated at home, while one patient developed severe infection with a fatal outcome and died due to severe bilateral pneumonia and acute respiratory distress syndrome (ARDS). Of the three MMN patients who recovered from COVID-19, in two cases there was no change in the condition of MMN, while one patient experienced mild worsening.

## Discussion

The main finding of this 6-year real-world follow-up study is that long-term immunotherapy in multifocal motor neuropathy (MMN) was associated with relative stability of motor disability, whereas fatigue, depressive symptoms, and several physical quality-of-life domains worsened over time. These findings suggest that the long-term burden of MMN extends beyond motor impairment alone and increasingly includes non-motor and psychosocial dimensions of the disease. To the best of our knowledge, this is the first real-world longitudinal study to evaluate these trajectories in MMN during a follow-up period that also encompassed the COVID-19 era. Previous studies, including our earlier cross-sectional work (Bozovic et al., 2019) and treatment-oriented clinical studies (Stangel et al., 2016; Harbo et al., 2009; Eftimov et al., 2009), provided important insight into therapeutic efficacy and quality of life, but did not address long-term changes in routine clinical practice.

Despite continuous immunotherapy, MMN remained associated with persistent functional impairment in our cohort. However, the absence of significant deterioration in disability scores suggests that long-term treatment may primarily contribute to stabilization rather than substantial functional recovery. This observation is in line with previous data demonstrating that immunoglobulin therapy improves strength and disability but does not fully prevent long-term disease burden (Stangel et al., 2016; Misbah et al., 2011; Harbo et al., 2009; Katzberg et al., 2016). These findings are consistent with the concept of MMN as a chronic, slowly progressive but treatable immune-mediated neuropathy (Lawson and Arnold, 2014).

Our results further confirm that physical domains remain more affected than mental domains in MMN, which is consistent with previous studies (Bozovic et al., 2019; Stangel et al., 2016). This predominance of physical impairment is expected given the motor-predominant nature of the disease and its impact on daily functioning and work ability. Nevertheless, the increase in fatigue and depressive symptoms observed in our cohort suggests that non-motor burden may accumulate over time even when overall mental health scores remain relatively preserved (Bozovic et al., 2017).

Interestingly, a non-significant improvement in I-RODS scores and an increase in general health and social functioning domains were observed. These findings may reflect long-term adaptation to chronic disease and the beneficial effects of continuous treatment and social support. In contrast, the worsening of bodily pain observed in our cohort may represent an emerging symptom burden that has been increasingly recognized in patients following COVID-19 infection (Şahin et al., 2021).

One of the most important longitudinal findings of our study is the significant increase in fatigue and depressive symptoms over time. Fatigue has previously been identified as a major contributor to disability in immune-mediated neuropathies (Merkies et al., 1999), while depressive symptoms have been shown to significantly affect quality of life in chronic inflammatory neuropathies (Bozovic et al., 2017). Our findings therefore support the growing recognition that MMN, although traditionally regarded as a purely motor disorder, carries a substantial non-motor burden that may increase over time. These findings are clinically relevant because they indicate that long-term patient burden in MMN may be underestimated if follow-up is focused exclusively on motor disability.

The follow-up period coincided with the COVID-19 pandemic, providing a unique opportunity to assess its multidimensional impact on patients with MMN. In our cohort, the pandemic was associated with disruption of regular neurological care, treatment interruption in a subset of patients, and substantial concern regarding its potential effect on disease course. These findings are consistent with previous reports in patients with chronic inflammatory neuropathies during the pandemic (Stojanov et al., 2021; Cascella et al., 2021). Broader evidence has also documented increased psychological burden in patients with autoimmune neurological disorders during the COVID-19 era (Taga and Lauria, 2022; Pugliatti et al., 2022). The observed increase in fatigue, depressive symptoms, and bodily pain in our cohort may therefore reflect the combined effect of chronic disease burden and pandemic-related psychosocial stressors, rather than a single causative factor. However, given the observational design and small sample size, the specific contribution of pandemic-related factors cannot be disentangled from the natural long-term course of the disease.

Taken together, our results suggest a shift in the long-term burden of MMN. While motor disability may remain relatively stable under long-term immunotherapy, non-motor symptoms such as fatigue and depression appear to increase over time and may increasingly shape patients’ perceived quality of life. These findings underscore the importance of comprehensive and multidisciplinary management strategies in MMN that extend beyond motor function alone. The absence of significant predictors in the multivariable regression model likely reflects the limited statistical power associated with the small sample size.

These findings have several practical implications for clinical care. Routine assessment of fatigue and depressive symptoms should be incorporated into standard follow-up of MMN patients, as these symptoms appear to increase despite motor stability. Early recognition of non-motor burden may enable timely psychological support, rehabilitation strategies and individualized treatment planning. A multidisciplinary approach involving neurologists, mental health professionals and rehabilitation specialists may therefore be essential for improving long-term outcomes in MMN.

### Study limitations

The present study has several limitations. First, the small sample size and single-center design reflect the rarity of MMN and limit the statistical power and generalizability of our findings. Second, a generic quality-of-life questionnaire (SF-36) was used, which is not disease-specific for MMN. The MMN-RODS scale was not available and validated at the time of baseline assessment and therefore could not be applied longitudinally. In addition, because no standardized instruments existed at the start of the study to evaluate the relationship between COVID-19 and inflammatory neuropathies, the pandemic-related impact was assessed using a conducted questionnaire.

Despite these limitations, this study provides the first longitudinal real-world evaluation of quality of life and disease outcome in patients with MMN during a six-year follow-up period that included the COVID-19 era.

## Conclusion

This six-year longitudinal follow-up provides novel real-world insight into the evolution of quality of life in patients with multifocal motor neuropathy during the COVID-19 era. Long-term immunotherapy appears to contribute primarily to stabilization of motor disability rather than full functional recovery. Despite this relative motor stability, fatigue and depressive symptoms increased significantly over time and appear to represent important contributors to reduced quality of life.

Our findings suggest that the long-term burden of MMN extends beyond motor impairment and progressively involves non-motor and psychosocial dimensions of the disease. Routine screening for fatigue and mental health symptoms should therefore become an integral part of clinical care in MMN. A multidisciplinary and holistic management approach may be essential for improving long-term patient outcomes. Incorporating simple, routine assessment of non-motor symptoms into follow-up visits may represent a feasible step toward improving long-term care and patient-centered management in MMN.

## Data Availability

The raw data supporting the conclusions of this article will be made available by the authors, without undue reservation.
